# TOPK Affects Autophagy of Skin Squamous Cell Carcinoma by Regulating NF-KB Pathway through HDAC1

**DOI:** 10.1155/2022/3771711

**Published:** 2022-06-15

**Authors:** Juan Li, Zhi-chao Zhang, Xiang-shi Yuan, Shan-shan Tang, Tao Wang, Hong-fu Liu, Yu Cao

**Affiliations:** ^1^Medical College of Soochow University, Suzhou 215123, China; ^2^Clinical Medical College, Guizhou Medical University, Guiyang 550004, China; ^3^Department of Dermatology and Venereal Diseases, Guiyang Center for Disease Control and Prevention, Guiyang, Guizhou 550000, China; ^4^Department of Occupational Health, Guiyang Center for Disease Control and Prevention, Guiyang, Guizhou 550000, China; ^5^Department of Immunization Planning, Guiyang Center for Disease Control and Prevention, Guiyang, Guizhou 550000, China; ^6^Department of General Surgery, The First Affiliated Hospital of Gannan Medical University, Ganzhou 341000, China

## Abstract

**Objective:**

To explore the effect and potential mechanism of T-LAK cell-originated protein kinase (TOPK) on autophagy in cutaneous squamous cell carcinoma (cSCC).

**Methods:**

Human cSCC cancer tissue and paracancerous tissue samples were collected clinically; immunohistochemistry was used to detect the expression of TOPK, nuclear factor *κ*B p65 (NF-*κ*B p65), phosphorylated nuclear factor *κ*B inhibitor *α* (p-I*κ*B*α*), Beclin-1, and microtubule-associated protein 1 light chain 3 (LC3) in cSCC tissue; and immunofluorescence was adopted to detect the coexpression of NF-*κ*B p65, p-I*κ*B*α*, and TOPK in cSCC tissue. After TOPK silencing or overexpression, SCL-1 and A431 cells were treated with PDTC and 3-MA, respectively. RT-qPCR and Western Blot were used to detect the mRNA and protein expressions of histone deacetylase 1 (HDAC1) in TOPK-silenced/overexpressing cells. Western Blot was performed to detect the protein expressions of NF-*κ*B p65, p-p65, I*κ*B*α*, p-I*κ*B*α*, Beclin-1, and LC3 in each group. Transwell and scratch healing experiments were used to detect the ability of cells to invade and migrate. The formation of autophagosomes in each group was observed by TEM.

**Results:**

Compared with adjacent tissues, TOPK, NF-*κ*B p65, p-I*κ*B*α*, Beclin-1, and LC3 were highly expressed in cSCC cancer tissues; TOPK and NF-*κ*B p65 were coexpressed; and TOPK and p-I*κ*B*α* were expressed in cSCC cancer tissues both increased. The mRNA and protein levels of TOPK in human cSCC cells were significantly higher than those in human normal skin HaCaT cells. After TOPK knockout, the expression of HDAC1, p-I*κ*B*α*/I*κ*B*α*, NF-*κ*B p65, p-p65, Beclin-1, LC3II/I proteins, cell invasion, and migration abilities were significantly reduced, and fewer autophagosomes were observed. Treatment with PDTC and 3-MA significantly downregulated NF-*κ*B pathway protein activity and autophagy level and reduced cell migration and invasion ability.

**Conclusion:**

TOPK promotes the malignant progression of cSCC by upregulating HDAC1 to activate the NF-*κ*B pathway and promote autophagy.

## 1. Introduction

Cutaneous squamous cell carcinoma (cSCC) is characterized by high incidence, strong invasion, and metastasis [1, 2]. Therefore, exploring the molecular mechanism affecting the occurrence and development of cSCC offers an important basis for targeted therapy of cSCC. Recent studies have shown that autophagy is related to the proliferation, apoptosis, and metastasis of cancer cells [3, 4]. Autophagy acts not only as a tumor suppressor but also as a cell survival mechanism promoting the growth of tumors, which is mainly related to tumor types [5, 6]. The regulatory mechanism of autophagy in cSCC and its influence on tumor are not completely clear.

T-LAK cell-originated protein kinase (TOPK) is a serine-threonine protein kinase, of which the overexpression is indicative of many cancers and tumor invasiveness [7–9]. There are few studies on cSCC, and its influence on autophagy is still unclear. Histone deacetylase, (HDAC) inhibitors are effective anticancer agents, which have shown curative effects on various human tumors, including cSCC [10]. According to reports, histone deacetylase 1 (HDAC1) is a member of HDAC family and an important epigenetic factor, which is related to the progress and prognosis of cancer [11, 12]. Recent studies have shown that TOPK can regulate the activity of HDAC1 [13], while HDAC1 can activate NF-*κ*B and lead to the malignant phenotype of glioblastoma [14]. Introduction of activated NF-*κ*B and HDAC1 generates gene silencing complexes that increase migration and invasion of prostate cancer cells [15]. In cSCC, activation of NF-*κ*B pathway can promote the expression of autophagy gene in transcription [16]. We found in previous studies that TOPK and autophagy proteins Beclin-1 and LC3 are highly expressed in cSCC clinicopathological paraffin sections and cSCC cells, and there is an interaction between TOPK and HDAC1. Therefore, we speculate that TOPK may activate autophagy and promote cSCC. In conclusion, this study mainly explored the effect of TOPK on autophagy in cSCC.

## 2. Materials and Methods

### 2.1. Pathological Sections of Cell Lines and cSCC

HaCaT cells from human normal skin andA431 and SCL-1 cells were bought from the Shanghai Enzymatic Research Biological Technology Co., Ltd., with the item numbers of CC-Y1177, CC-Y1033, and CC-Y1669, all of which were cultured in DMEM containing 10% fetal bovine serum, 100 U/mL penicillin, and 100 mg/mL streptomycin in an incubator at 37°C and 5% CO2.

Pathological sections of human cSCC cancer tissues and adjacent tissues were obtained from the Department of Pathology of First Affiliated Hospital of Gannan Medical University and the Department of Dermatology of Affiliated Hospital of Guizhou Medical University. All tissue sections have been confirmed by the Department of Pathology, and the collection of pathological sections has been approved by the hospital ethics committees.

### 2.2. Immunohistochemistry (IHC)

The tissue specimens were fixed with 4% paraformaldehyde, embedded in paraffin, and sectioned continuously at 4 to 5 *μ*m. After dewaxing and repairing with high-pressure antigen, samples were incubated with NF-*κ*B p65 (ab288751, Abcam), Beclin-1 (ab207612, Abcam), TOPK (ab280209, Abcam), LC3 (ab128025, Abcam), and p-I*κ*B*α* (sc-8404, Santa Cruz Biotechnology) at 4°C overnight. Subsequently, Rabbit Anti-Human IgG H&L antibody (ab6759, Abcam) was used as the secondary antibody. DAB staining, hematoxylin restaining, hydrochloric alcohol differentiation, and blue return were added. Finally, the sections were dehydrated, hyalinized, sealed, and microscopically examined. The target protein staining was in brown, and the nucleus was in blue-purple.

### 2.3. Immunofluorescence Assay

Paraffin sections were dewaxed and repaired with high pressure antigen, and then 3% hydrogen peroxide was added to remove endogenous enzymes. After blocking with normal fetal bovine serum, NF-*κ*B p65 (ab288751, Abcam), p-I*κ*B*α* (sc-8404, Santa Cruz Biotechnology), and TOPK (ab280209, Abcam) antibodies were incubated overnight at 4°C. Anti-Human IgG1 H&L (Alexa Fluor® 488) (ab200622, Abcam) or Goat Anti-Human IgG (H + L) Cross-Adsorbed Secondary Antibody, Alexa Fluor™ 594 (A-11014, Invitrogen) were used as secondary antibodies. The nuclei were stained with DAPI (Invitrogen). The fluorescence was observed under IX73 inverted fluorescence microscope (Olympus Company, Japan).

### 2.4. Real-Time Fluorescence Quantitative PCR (RT-qPCR)

A431 and SCL-1 cells as well as human normal skin HaCaT cells were selected. Total RNA was separated from cells by TRIzol reagent, and the concentration and purity of total RNA were detected by NanoDrop spectrophotometer. PrimeScript™ RT kit was used to synthesize cDNA, and the cDNA was taken as template for detection by fq-PCR. With GAPDH as internal reference, 2^-△△Ct^ method was used for relative quantitative analysis. Primer sequences were summarized in Table 1.

### 2.5. Western Blot

Total proteins in human cSCC cell lines (A431 and SCL-1 cells) and human normal skin HaCaT cells were extracted by RIPA lysate, and the concentrations of the proteins were calculated by BCA protein concentration determination kit. Protein samples were separated by electrophoresis before transferring to PVDF membrane. The membrane was incubated in 5% skim milk at indoor temperature for 2 h, followed by incubation with primary antibodies at 4°C overnight. Then, the membrane was incubated with HRP-coupled secondary antibody (ab6759, Abcam) for 2 h at room temperature. ECL was developed and exposed in gel imaging system. The gray value of band was measured by Image-Pro Plus 6.0 software with GAPDH as reference. The relative expression level of the target band was obtained by the gray ratio with the internal reference.

### 2.6. Cell Grouping and Treatment

SCL-1 and A431 cells were transfected with TOPK interference and overexpression plasmids. The mRNA and protein expression of TOPK in the cells were measured by RT-qPCR and Western Blot to verify the transfection efficiency. Three siRNAs of TOPK were designed and transfected into A431 cells. There was a total of 8 groups: SCL-1 control group, SCL-1+ si TOPK NC group (TOPK-siRNA negative control vector transfected into SCL-1 cells), SCL-1+TOPK si TOPK group (TOPK-siRNA transfected into SCL-1 cells), A431 control group, A431+ TOPK OE NC group (pcDNA3.1-TOPK negative control empty vector plasmid transfected into A431 cells), A431+TOPK OE group (pcDNA3.1-TOPK plasmid transfected into A431 cells), A431+TOPK OE+PDTC group (cells continued to be cultured in PDTC medium for 48 h after successful transfection), and A431+TOPK OE+3-MA group (cells continued to be cultured in 3-MA medium for 48 h after successful transfection). RT-qPCR and Western Blot were conducted to measure transfection efficiency, and RT-qPCR was carried out to test the mRNA and protein expression of HDAC1 in TOPK silenced/overexpressed cells.

### 2.7. Transwell

After collecting and centrifuging the transfected cells in each group, cells were resuspended in serum-free culture medium. The number of cells in each chamber was 50,000. Complete medium (500 *μ*L) was added to the lower part of the invasion chamber, and serum-free medium (300 *μ*L) was added to upper chamber, followed by incubating the cells in 5% CO_2_ at 37°C. After 48 h, the cells on the upper chamber surface were removed with cotton swabs. After washing with PBS, the cells adhered to the lower chamber membrane were fixed with 4% paraformaldehyde for 15 min, stained using 0.1% crystal violet solution, and photographed and observed to evaluate the invasion ability of the cells.

### 2.8. Scratch Healing Test

Transfected cells in each group were collected and spread. Each hole was scratched with a 200 *μ*L gun head when the density reaches over 90%. After discarding the culture medium, PBS was used for washing for 3 times, and pictures of scratches in each hole were taken after changing to serum-free medium; the cells were placed in an incubator, and the scratches of each hole were photographed again after 48 h. The width of scratches was measured, and then the migration rate of cells was calculated. The formula migration rate = migration distance/migration time.

### 2.9. Observation of Autophagy Corpuscles by Transmission Electron Microscope

Transfected cells in each group were fixed by 2.5% glutaraldehyde, rinsed with 0.1 M PBS, fixed with 1% acid, and rinsed with 0.01 M PBS. After dehydration by gradient ethanol and acetone, it stayed overnight with pure embedding solution at room temperature. The cell mass was put into the embedding mold. After adding the embedding agent, the liquid was turned into solid by heating (oven). The solid was sectioned with an ultrathin slicer. Then, the sections were stained using 3% uranium acetate-lead citrate before TEM observation.

### 2.10. Statistical Analysis

The statistical analysis was performed using SPSS 20.0. All experiments were conducted in triplicate, and the quantitative results were expressed by the mean ± standard deviation (SD). The difference between two groups was tested by *T* test, and difference between multiple groups was tested by one-way ANOVA, and the pairwise comparison adopts S-N-K method. The two-sided *P* < 0.05 was indicated as significant. The statistical chart was drawn by GraphPad Prism 6.

## 3. Results

### 3.1. High Expression Levels of TOPK, NF-*κ*B p65, p-I*κ*B*α*, Beclin-1, and LC3 in cSCC Cancer Tissues

**Figure 1:**
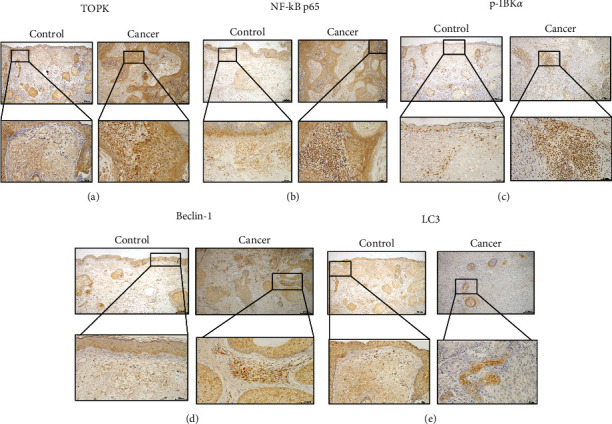
Expressive level of TOPK, NF-*κ*B p65, p-I*κ*B*α*, Beclin-1, and LC3 in cSCC cancer tissues and adjacent tissues. IHC analysis revealed that the expression of TOPK (a) was higher in cSCC cancer tissues than in adjacent tissues. The expressive levels of NF-*κ*B p65 (b), p-I*κ*B*α* (c), Beclin-1 (d), and LC3 (e) were significantly higher in cSCC cancer tissues than in adjacent tissues.

IHC analysis revealed that the expression of TOPK was higher in cSCC cancer tissues than that in the adjacent tissues (Figure 1(a)). Meanwhile, the expressive levels of NF-*κ*B p65, p-I*κ*B*α*, Beclin-1, and LC3 were significantly higher in cSCC cancer tissues than those in adjacent tissues (Figures 1(b)–1(e)).

### 3.2. Coexpression Levels of TOPK, NF-*κ*B p65, and p-I*κ*B*α* in cSCC Cancer Tissues

**Figure 2:**
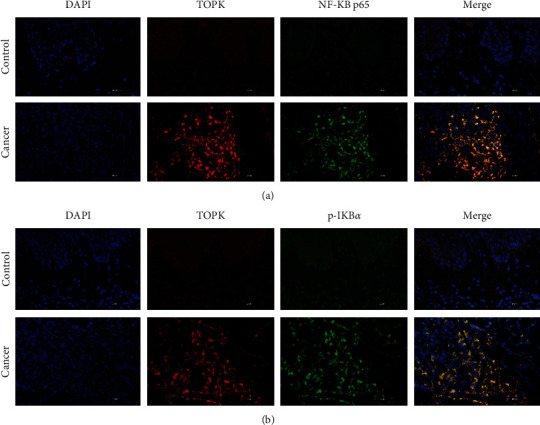
Expression level of TOPK and NF-*κ*B p65 and p-I*κ*B*α* in cSCC cancer tissues and adjacent tissues. Immunofluorescence results showed that TOPK and NF-*κ*B p65 (a) and TOPK and p-I*κ*B*α* (b) were highly expressed in cSCC cancer tissues.

Immunofluorescence results showed that TOPK and NF-*κ*B p65, TOPK, and p-I*κ*B*α* were highly expressed in cSCC cancer tissues (Figure 2), and these proteins were mainly located around and in the nucleus.

### 3.3. mRNA and Protein Expression Level of TOPK in cSCC Cells

**Figure 3:**
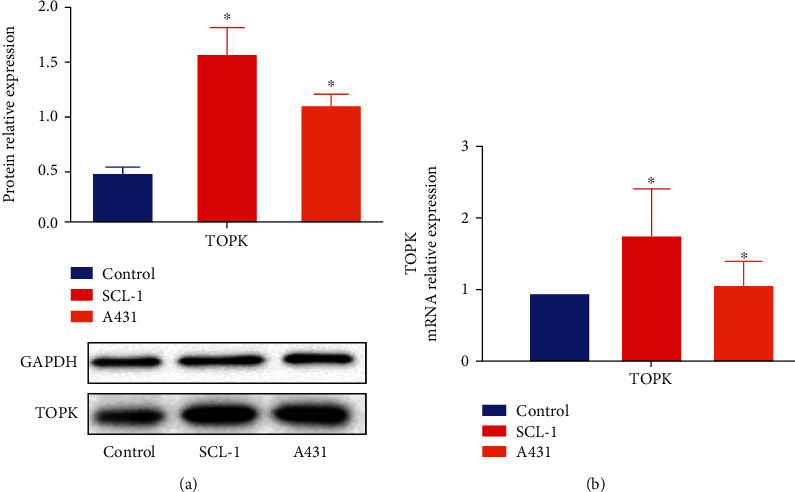
mRNA and protein expression level of TOPK in cSCC cells. RT-qPCR and Western Blot revealed that the protein (a) and mRNA (b) expression levels of TOPK were significantly higher in human cSCC cells than in human HaCaT cells.

RT-qPCR and Western Blot revealed that the mRNA and protein expression levels of TOPK were significantly higher in human cSCC cells than that in HaCaT cells (*P* < 0.05, Figures 3(a) and 3(b)).

### 3.4. Protein Expression and mRNA of TOPK in Transfected Cells

**Figure 4:**
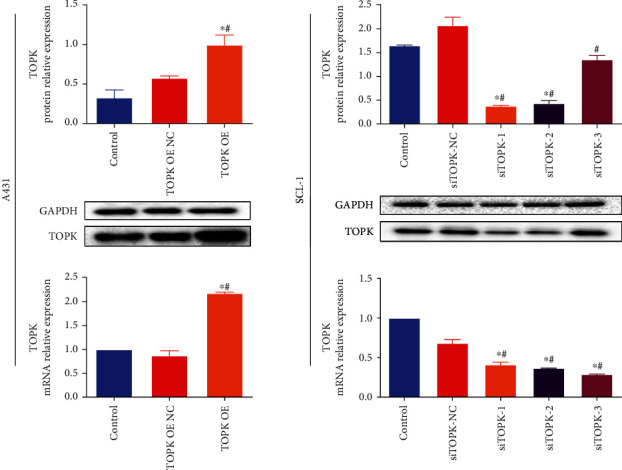
mRNA and protein expression level of TOPK in cells after transfection. RT-qPCR and Western Blot revealed that in A431 and SCL-1 cells, compared with the control and no-load groups, TOPK expression was significantly increased in TOPK OE group (*P* < 0.05) while significantly decreased in siTOPK-1/2/3 group (*P* < 0.05), indicating that TOPK overexpression and interference transfection were successful; siTOPK-1 was selected for follow-up experiment. ^∗^*P* < 0.05 vs. control and ^#^*P* < 0.05 vs. TOPK OE NC or siTOPK-NC.

RT-qPCR and Western Blot revealed that in A431 and SCL-1 cells, compared with the control groups, TOPK expression was significantly increased in TOPK OE group (*P* < 0.05,Figures 4(a) and 4(b)), while significantly decreased in siTOPK-1/2/3 group (*P* < 0.05,Figures 4(c) and 4(d)), indicating that TOPK overexpression and interference transfection were successful. The siTOPK-1 was selected for follow-up experiment.

### 3.5. The Effect of TOPK Silencing/Overexpression on mRNA and Protein Expression Level of HDAC1 in Cells

**Figure 5:**
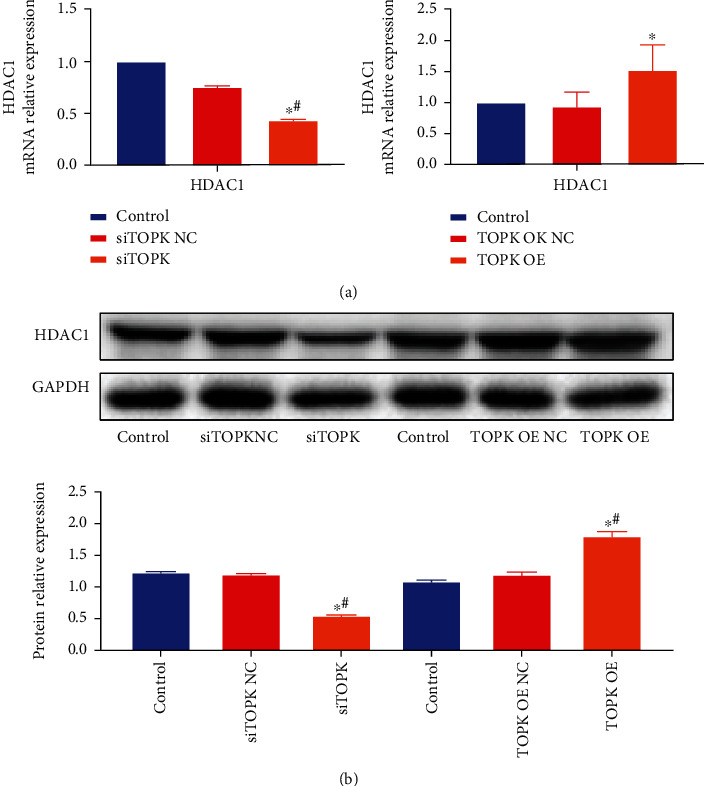
The influence of TOPK silencing/overexpression on cell HDAC1 mRNA and protein expression. Compared with the control and no-load groups, the mRNA and protein expression of HDAC1 decreased significantly in siTOPK group (*P* < 0.05) while increased significantly in TOPK OE group (*P* < 0.05). See Figures 5(a) and 5(b). ^∗^*P* < 0.05 vs. control and ^#^*P* < 0.05 vs. TOPK OE NC or siTOPK-NC.

As shown in Figure 5, compared with the control groups, the mRNA and protein expression of HDAC1 decreased significantly in siTOPK group (*P* < 0.05) while increased significantly in TOPK OE group (*P* < 0.05).

### 3.6. The Effect of TOPK Silencing/Overexpression on the Protein Expression Levels of NF-*κ*B p65, p-p65, I*κ*B*α*, and p-I*κ*B*α* in cells

**Figure 6:**
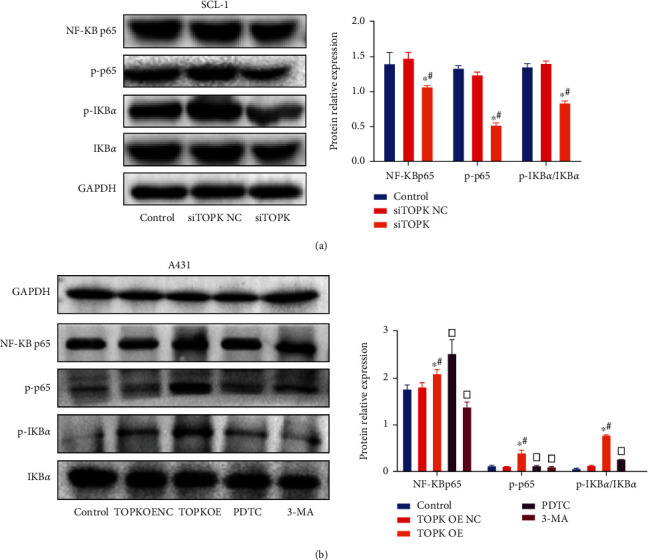
The effect of TOPK silencing/overexpression on cell NF-*κ*B p65, p-p65, I*κ*B*α*, and p-I*κ*B*α* protein expression. Compared with the control and no-load groups, the protein expression level of p-I*κ*B*α*/I*κ*B*α*, NF-*κ*B p65, and p-p65 decreased significantly, and SCL-1 cells of siTOPK group decreased significantly (*P* < 0.05) but increased significantly in A431 cells of TOPK OE group (*P* < 0.05); compared with TOPK OE group, the protein expression level of p-I*κ*B*α*/I*κ*B*α* and p-p65 decreased significantly in PDTC and 3-MA groups (*P* < 0.05). See Figure 6(a) and 6(b). ^∗^*P* < 0.05 vs. control, ^#^*P* < 0.05 vs. TOPK OE NC or siTOPK-NC, and ^□^*P* < 0.05 vs. TOPK OE.

As shown in Figure 6, compared with the control and no-load groups, the protein expression level of p-I*κ*B*α*/I*κ*B*α*, NF-*κ*B p65, and p-p65 decreased significantly, and SCL-1 cells of siTOPK group decreased significantly (*P* < 0.05) but increased significantly in A431 cells of TOPK OE group (*P* < 0.05); compared with TOPK OE group, the protein expression level of p-I*κ*B*α*/I*κ*B*α* and p-p65 decreased significantly in PDTC and 3-MA groups (*P* < 0.05).

### 3.7. The Effect TOPK Silence/Overexpression on Cell Invasion and Migration Ability

**Figure 7:**
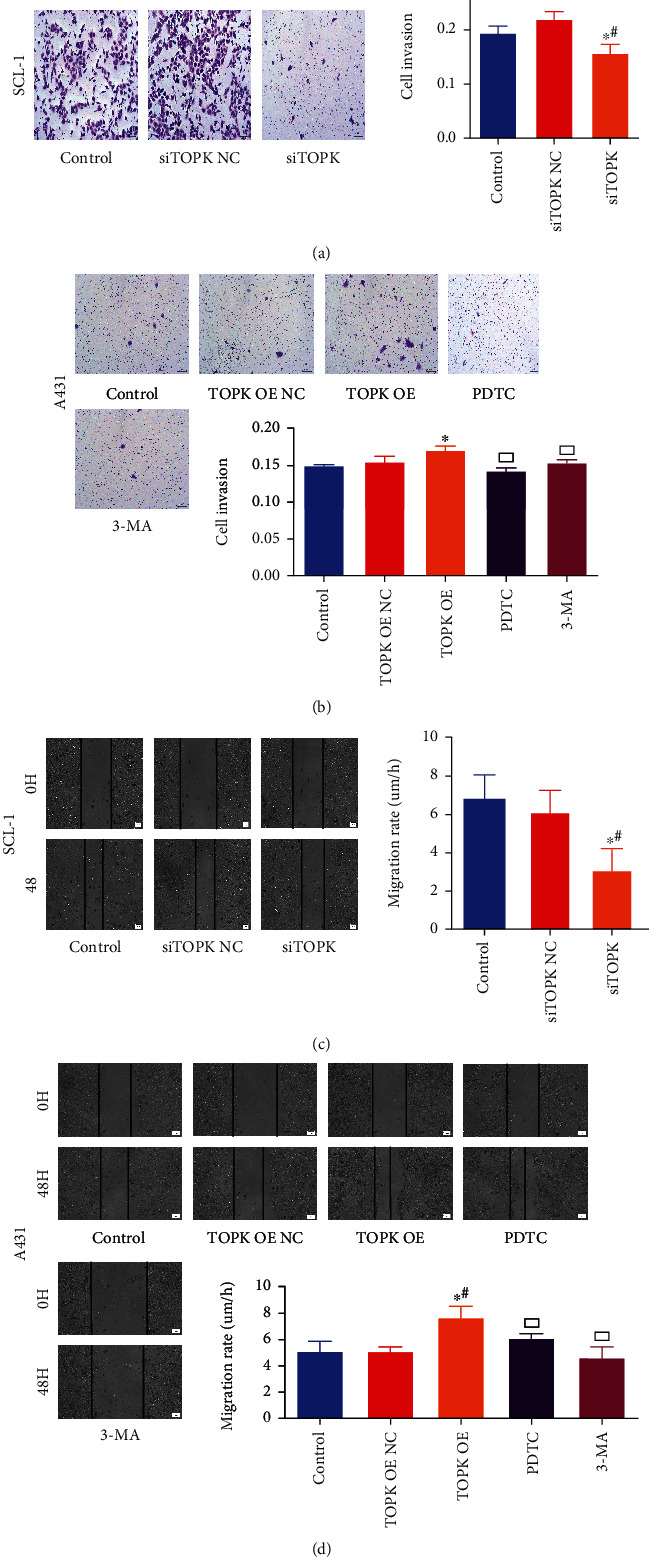
The effect of TOPK silencing/overexpression on cell invasion and migration. Compared with the control and no-load groups, the invasion and migration ability decreased significantly in SCL-1 cells of siTOPK group (*P* < 0.05) while increased significantly in A431 cells of TOPK OE group (*P* < 0.05). Compared with TOPK OE group, the invasion and migration ability of A431 cells decreased significantly in PDTC group and 3-MA group (*P* < 0.05). ^∗^*P* < 0.05 vs. control, ^#^*P* < 0.05 vs. TOPK OE NC or siTOPK-NC, and ^□^*P* < 0.05 vs. TOPK OE.

As shown in Figure 7, compared with the control groups, the invasion and migration ability decreased significantly in SCL-1 cells of siTOPK group (*P* < 0.05) while increased significantly in A431 cells of TOPK OE group (*P* < 0.05). Compared with TOPK OE group, the invasion and migration ability of A431 cells decreased significantly in PDTC group and 3-MA group (*P* < 0.05).

### 3.8. The Effect TOPK Silencing/Overexpression on Autophagy and Protein Expressions of Beclin-1 and LC3

**Figure 8:**
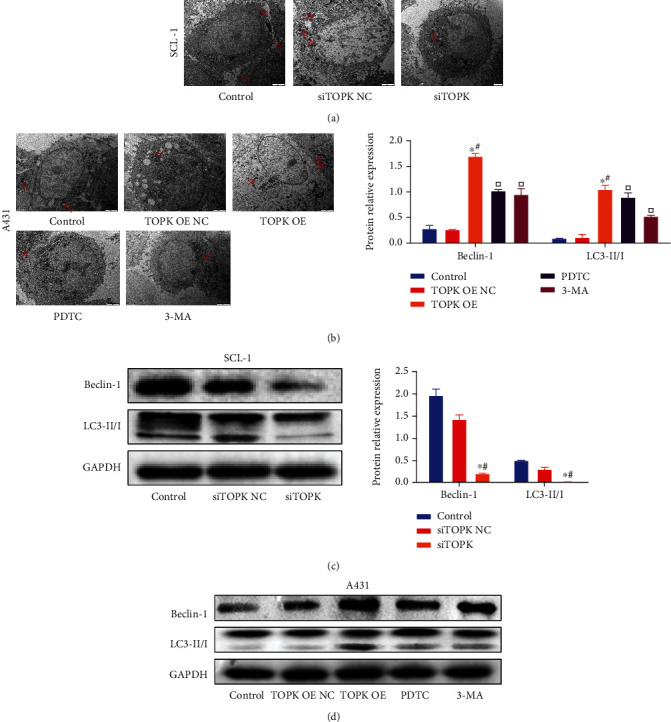
The effect of TOPK silencing/overexpression on autophagy. The autophagosomes were observed by TEM. Compared with the control and no-load group S, the autophagosomes decreased in SCL-1 cells of siTOPK group while increased in A431 cells of TOPK OE group. Compared with TOPK OE group, the number of autophagy bodies decreased in PDTC group and 3-MA group; Western Blot revealed that the expressions of Beclin-1 and LC3II/I decreased significantly in SCL-1 cells of siTOPK group (*P* < 0.05) and increased significantly in A431 cells of TOPK OE group (*P* < 0.05); compared with TOPK OE group, the protein expression levels of Beclin-1 and LC3II/I decreased significantly in PDTC group and 3-MA group (*P* < 0.05). See Figure 8(c) and 8(d). ^∗^*P* < 0.05 vs. control, ^#^*P* < 0.05 vs. TOPK OE NC or siTOPK-NC, and ^□^*P* < 0.05 vs. TOPK OE.

The autophagosomes were observed by TEM. Compared with the control group, the autophagosomes decreased in SCL-1 cells of siTOPK group while increased in A431 cells of TOPK OE group. Compared with TOPK OE group, the number of autophagy bodies decreased in PDTC group and 3-MA group; Western Blot revealed that the expressions of Beclin-1 and LC3 II/I decreased significantly in SCL-1 cells of siTOPK group (*P* < 0.05) and increased significantly in A431 cells of TOPK OE group (*P* < 0.05); compared with TOPK OE group, the protein expression levels of Beclin-1 and LC3 II/I decreased significantly in PDTC group and 3-MA group (*P* < 0.05, Figure 8).

## 4. Discussion

It was found that TOPK was limited in normal skin tissues but was highly expressed in cSCC cells and cancer tissues [17, 18]. Knocking down the expression of TOPK in cSCC cells by small molecule interference technology significantly reduced the invasion and metastasis ability of cancer cells, while upregulating TOPK expression could further promote malignant progress; this is consistent with other scholars' reports [7–9], suggesting that TOPK can acts as potential target in treatment of cSCC.

Autophagy affects the malignant progression of cSCC, and autophagy activity is related to cSCC invasiveness [19]. It has been reported that autophagy-related Beclin-1 is overexpressed in cSCC lymph node metastasis [20]. In this study, we found that the expressions of Beclin-1 and LC3 were significantly higher in cSCC cancer tissues than in adjacent tissues, which was consistent with the expression trend of TOPK. Therefore, we put forward our hypothesis: TOPK may activate autophagy and promote cSCC. And the relationship between TOPK and autophagy in cSCC was also discussed. It was observed that the autophagy level decreased after knocking out the expression of TOPK in cSCC cells but increased after upregulating the expression of TOPK; on the basis of upregulating TOPK, the autophagy level of cells was significantly inhibited after adding autophagy inhibitor 3-MA, and the migration and invasion ability of cells also decreased; this suggests that TOPK could activate autophagy and promote the metastasis of cSCC cells, which is in line with the report of Yoshihara et al. [21] who found that autophagy is activated during the disease progression of cSCC patients. However, there are few studies on the correlation between TOPK and autophagy. According to report on glioma, TOPK inhibits autophagy of glioma cells in glioma, thus enhancing the sensitivity of glioma cells to temozolomide [22] and promoting apoptosis of glioma cells. This also suggests that the regulation of autophagy by TOPK is influenced by tumor types.

On the basis of the existing research, we deeply discussed the molecular mechanism of TOPK regulating autophagy. The method of protein interaction analysis such as immunoprecipitation and mass spectrometry identification was used to explore how TOPK can promote tumor by regulating autophagy. Mass spectrometry results showed that TOPK could bind HDAC1 protein. The expression of HDAC1 significantly increased after transfecting TOPK overexpression vector into A431 cells, while the expression of HDAC1 significantly decreased after transfecting TOPK interference vector into SCL-1 cells. TOPK can upregulate the expression of HDAC1. HDAC1 can interact with NF-*κ*B to promote tumor migration and invasion [14, 15, 23, 24]. Abnormal activation of NF-*κ*B pathway can cause skin diseases such as psoriasis and cSCC [25]. Previous studies [16] showed that activating NF-*κ*B pathway in cSCC can promote autophagy gene expression. Therefore, we speculate that TOPK may regulate the activation of NF-*κ*B through HDAC1, thus activating the protein expression of downstream autophagy. To verify this hypothesis, we first detected the expression levels of NF-*κ*B p65 and p-I*κ*B*α* in cSCC tissue samples by immunohistochemistry. The results showed that NF-*κ*B p65 and p-I*κ*B*α* were significantly higher in cancer tissues. Further immunofluorescence results revealed that the coexpression of TOPK/NF-*κ*B p65 and TOPK/p-I*κ*B*α* increased in cSCC cancer tissues; then, HDAC1 and NF-*κ*B p65 activation-related proteins decreased in cSCC cells with knocking down TOPK, while overexpression of TOPK showed opposite effects. On the basis of overexpression of TOPK, after adding NF-*κ*B inhibitor PDTC, the protein activity of NF-*κ*B pathway and autophagy level decreased, so did the migration and invasion ability of cells. This suggests that TOPK may affect the activity of NF-*κ*B pathway through HDAC1 and then affect the occurrence of autophagy. The specific regulatory mechanism will be discussed in detail in the next step.

In conclusion, TOPK highly expressed in cSCC can activate the NF-*κ*B pathway by upregulating HDAC1 and promote autophagy to aggravate the progression of cSCC. This study provides a theoretical basis for the targeted therapy of cSCC, indicating that TOPK regulation of autophagy may be a promising therapeutic target for cSCC, and provides a new idea for the individualized treatment of cSCC in the future.

## Figures and Tables

**Table 1:** List of primers used in this study.

Gene	Primer sequence	Product length (bp)
TOPK F	TACTATGCAGCGTTGGGAACT	122
TOPK R	GCAGAAGGACGATCTTTAGGGTC	
GAPDH F	TGGCACCCAGCACAATGAA	186
GAPDH R	CTAAGTCATAGTCCGCCTAGAAGCA	
HDAC1 F	CATCTCCTCAGCATTGGCTT	126
HDAC1 R	TATTATGGACAAGGCCACCC	

## Data Availability

Data will be available from the corresponding author upon request.
